# Supplementary material for: Optimization of Coffee Oil Extraction from Defective Beans Using a Supercritical Carbon Dioxide Technique: Its Effect on Volatile Aroma Components
Source: Foods. 2023 Jun 28;12(13):2515. doi: 10.3390/foods12132515 (PMC10340388; doi:10.3390/foods12132515)
Supplement: Supplementary file 1 [file foods-12-02515-s001.zip › foods-2474705-supplementary.pdf]

foods MDPI

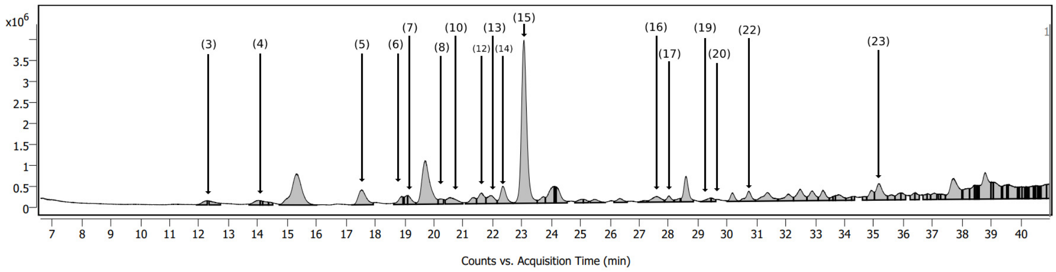

Figure S1. Chromatograms of volatile aroma compounds of coffee oil obtained using the DSE method. Peak numbers correspond to those of Table 9.
